# VASH-1 Regulates Oxidative Stress and Fibrosis in Diabetic Kidney Disease via SIRT1/HIF1α and TGFβ1/Smad3 Signaling Pathways

**DOI:** 10.3389/fmolb.2020.00137

**Published:** 2020-07-10

**Authors:** Huiwen Ren, Ying Shao, Can Wu, Chuan Lv, Yang Zhou, Qiuyue Wang

**Affiliations:** ^1^Department of Endocrinology, The First Affiliated Hospital of China Medical University, Shenyang, China; ^2^Advanced Institute for Medical Sciences, Dalian Medical University, Dalian, China; ^3^Department of Endocrinology, The Second Affiliated Hospital of China Medical University, Shenyang, China; ^4^Department of Gastroenterology and Endoscopy, The First Affiliated Hospital of China Medical University, Shenyang, China; ^5^Department of Endocrinology, The People's Hospital of Liaoning Province, Shenyang, China

**Keywords:** vasohibin-1, oxidative stress, fibrosis, diabetic kidney disease, diabetes

## Abstract

**Aims:** To investigate the role of Vasohibin-1 (VASH-1), silence information adjustment factor 2-related enzyme 1 (SIRT1)/hypoxic-inducible factor 1α (HIF1α) and transforming growth factor-β1 (TGFβ1) /Smad3 signaling pathways in oxidative stress and fibrosis of diabetic kidney disease (DKD).

**Materials and Methods:** A diabetic rat model was established *in vivo* and rat mesangial cells (RMCs) were cultured *in vitro* with high glucose via transfection with *Vash1* small interfering RNA (siRNA), *Hif1a* siRNA, *Sirt1* siRNA and TGFβ1/Smad3 pathway inhibitor (SB431542). Renal histology was used to detect renal changes. Real-time PCR and western blot were used to analyze the expression of VASH-1, SIRT1, HIF1α, TGFβ1, Smad3, vascular endothelial growth factor (VEGF), connective tissue growth factor (CTGF) and fibronectin (FN). Expression levels of tumor necrosis factor-α (TNFα), TGFβ1, superoxide dismutase (SOD), catalase (CAT), glutathione peroxidase (GSH-PX), and malondialdehyde (MDA) in rat tissues and cell culture supernatant were detected by ELISA and chemiluminescence assay, while cell proliferation was detected by CCK-8.

**Results:** The level of VASH-1 in renal tissues of diabetic rats was decreased, while both high glucose and *Vash1* siRNA inhibited the expression of VASH-1 and SIRT1, increased the levels of HIF1α, TGFβ1, and Smad3 in RMCs, thus up-regulating oxidative stress and fibrosis factors, and abnormally increasing cell proliferation activity (*P* < 0.05). However, inhibition of SIRT1/HIF1α signaling pathway only reduced TGFβ1 and Smad3 (*P* < 0.05), while VASH-1 remained unchanged (*P* > 0.05).

**Conclusion:** VASH-1 was under-expressed in renal tissues of diabetic rats and regulated the pathological process of oxidative stress and fibrosis in DKD via downstream SIRT1/HIF1α and TGFβ1/Smad3 signaling pathways.

## Highlights

- VASH-1 expression is suppressed in renal tissues of diabetic rats regulating VEGF, CTGF, and FN.
- VASH-1 can regulate the pathological process of oxidative stress and fibrosis in DKD via SIRT1/HIF1α and TGFβ1/Smad3 pathway.

## Introduction

Diabetic kidney disease (DKD) is the most common cause of end-stage renal disease (ESRD), clinical manifestations of which are characterized by slow development of continuous proteinuria, eventually leading to renal failure, and pathological features such as glomerular basement membrane thickening, glomerular hypertrophy, abnormal proliferation of glomerular mesangial cells and deposits of extracellular matrix (ECM), and even glomerular and interstitial fibrosis. Oxidative stress and fibrosis are two of important pathological changes in DKD (Affara et al., 2013; Afkarian et al., 2016).

Vasohibin-1 (VASH-1) is an emerging anti-angiogenic factor produced and secreted by endothelial cells with the target protein, vascular endothelial growth factor (VEGF) (Akgun and Ertel, 1985). Recent reports show that VASH-1 is present in glomeruli mesangial cells as well (Bergers and Hanahan, 2008), and VASH-1 also plays a potential protective role in kidney diseases via negative feedback (Brownlee et al., 2016). One recent study has indicated that silence information adjustment factor 2-related enzyme 1 (Sirtuin 1, SIRT1) and superoxide dismutase 2 (SOD2) are also two important downstream targets for VASH-1 (Chen et al., 2015). VASH-1 was also shown to play a possible anti-fibrosis role through the transforming growth factor β1 (TGFβ1) /Smad3 pathway (Didion and Faraci, 2005; Dei Cas and Gnudi, 2012). However, the specific mechanism of VASH-1 regulation for these downstream targets has not been clarified.

Here, we detected kidney VASH-1 levels in diabetic rats induced by STZ *in vivo*, and levels of oxidative stress and fibrosis factors in high glucose and *Vash1* siRNA cultured rat mesangial cells (RMCs) *in vitro* to investigate the function of VASH-1 with SIRT1/HIF1α and TGFβ1/Smad3 pathway regulating oxidative stress and fibrosis in DKD.

## Materials and Methods

### Establishment of Diabetic Rat Model

Sprague-Dawley (SD) male rats (SPF grade, 8 weeks, 180–220 g), were purchased from Beijing Vital River Laboratory Animal Technology Co., Ltd. Animals were housed in a standard specific pathogen free (SPF) laboratory at room temperature 23 ± 2°C, 12 h light/dark cycle, humidity 55 ± 5%. They were housed three to a cage with free access to water and food. All the experiments were carried out between 9 and 11 am in order to avoid circadian rhythm-induced changes. The experimental protocols of all animals were approved by the Institutional Animal Care and Use Committee (IACUC) of the First Affiliated Hospital of China Medical University (Approval no-2017043).

We used a well-established high-fat diet (D12492, Rodent Diet with 60% fat, 20% carbohydrate, and 20% protein, total 5.24 kcal/gm, Research Diets, Inc., USA) and low dose intraperitoneal injection streptozotocin (STZ, 35 mg/kg, cold 0.1 M citrate buffer pH 4.5, S0130, Sigma-Aldrich, USA) to establish rat diabetes model with diabetes mellitus group (DM, *n* = 6 rats) (Hasegawa et al., 2013; Franceschi and Campisi, 2014). Meanwhile, rats of the same age, fed with a control diet (D12450J, Rodent Diet with 10% fat, 70% carbohydrate, 20% protein, total 3.85 kcal/gm, Research Diets, Inc., USA), were selected as a normal control group (NC, *n* = 6 rats) (Hinamoto et al., 2014).

Intra-peritoneal glucose tolerance test (IPGTT) and insulin resistance test (IRT) was conducted in the 16th and 32nd week (16 and 32 W), and Graphpad software was used to calculate the area under curve of blood glucose (AUC GLU), serum insulin (AUC INS) and ratio (AUC ratio). Homeostasis model assessment of insulin resistance (HOMA-IR, fasting plasma glucose[FPG] [mmol/L] × fasting insulin [FINS] [mIU/L]/22.5) and insulin sensitive index (ISI, -ln [FPG × FINS]) were measured. A metabolic cage was then used to collect urine to detect microalbunminuria (MAU, CSB-E12991r, CUSABIO, Wuhan, China) and urine creatinine (uCr, C011-2, Nanjing Jiancheng Bioengineering Institute, China), then 24 h urine volume (24 h UV) was calculated. Urinary albumin to creatinine (UACR, MAU/uCr, mg/g) was used for the evaluation of urine protein (Hosaka et al., 2009).

### Renal Histological Examination

At the end of the experiment, all the animals were anesthetized and sacrificed with isoflurane. Kidney tissues were perfused by cold normal saline then resected and stored in 4% paraformaldehyde solution for further study. Fixed renal tissues were trimmed longitudinally and routinely processed. Tissue processing was done with dehydration in ascending grades of alcohol, clearing in xylene and embedded in paraffin wax. Paraffin wax embedded tissue blocks were sectioned at 5 μm thickness with a Rotary Microtome. All kidney slides were stained with Hematoxylin & Eosin (HE) stain, Periodic Acid-Schiff (PAS) stain and Masson stain. VASH-1 and VEGF in kidney were observed with immunofluorescence methods. The prepared slides were examined under microscope at 400 times magnification.

### Cell Culture

The RMC cell line (HBZY-1, a rat mesangial cell line) was purchased from American Type Culture Collection (ATCC, USA). They were inoculated into 25 cm^2^ culture flasks and cultured in D-MEM medium (Life Technologies, Carlsbad, CA, USA) supplemented with 10% fetal bovine serum (FBS, Gibco, USA) after thawing. Cells in their 5^th^ to 9^th^ generation of logarithmic growth phase were then incubated at 37°C with saturated humidity, in 5% CO_2_ and 95% air. Cell concentrations were 2 × 10^6^/2 ml in 25 cm^2^ cell culture flasks for western blot and 5 × 10^5^/2 ml in a 6-well tissue culture plates for Real-time PCR and ELISA. When the degree of cell fusion reached 70–80%, cells were synchronized after starvation with Opti-MEM (Gibco, USA) without FBS overnight, and then were used for subsequent experiments.

Normal glucose (5.5 mmol/L glucose), high mannitol (5.5 mmol/L glucose+24.5 mmol/L mannitol, osmotic pressure control) and high glucose (30 mmol/L glucose) were used to culture RMCs, respectively, and were collected after 12, 24, 48, and 72 h culture, then stored at −80°C.

### Transient Transfections and Pathway Inhibitor

In order to ensure the effectiveness of transfection, we used small interfering RNA (siRNA) for silencing of rat *Vash1* mRNA (GenBank no. NM_503052) designed and synthesized by GenePharma (Shanghai, China). According to the instructions of the transfection reagent, three different sequences (siRNA-V1, V2, and V3, Table 1) and non-specific siRNA (siRNA-V) were used as controls. Cells were plated into six-well plates and after 24 h starvation synchronization with Opti-MEM, they were transfected with 100 pmol/4 μg siRNA. Meanwhile, RMCs were also transiently transfected with *Sirt1* siRNA or *Hif1a* siRNA (sc-108043 or sc-45919, Santa Cruz, CA) by Lipofectamine 2000 (Invitrogen, USA), based on our previous research (Huang et al., 2014). Six hours after transfection, cells were treated with normal or high glucose media. Moreover, cells were cultured with a signal inhibitor to suppress rat TGFβ1/Smad3 pathway. The inhibitor is called SB431542 (10 μmol/L, #14775) and was designed as well as synthesized by Cell Signaling Technology as described in previous studies (Kanwar et al., 2011; Kanomata et al., 2013).

**Table 1:** *Vash1* siRNA gene sequences.

**Gene**	**Sequences**	
*Vash1* siRNA	siRNA-V1 Forward	5' - CCC AAG AUU CCC AUA CCA ATT - 3'
(NM_503052)	siRNA-V1 Reverse	5' - UUG GUA UGG GAA UCU UGG GTT - 3'
	siRNA-V2 Forward	5' - GCC AUU CAA AUG CCU GGA ATT - 3'
	siRNA-V2 Reverse	5' - UUC CAG GCA UUU GAU UGG CTT - 3'
	siRNA-V3 Forward	5' - GGA CCG GAA GAA GGA UGU UTT - 3'
	siRNA-V3 Reverse	5' - AAC AUC CUU CUU CCG GUC CTT - 3'
	NC-siRNA-V Forward	5' - UUC UCC GAA CGU GUC ACG UTT - 3'
	NC-siRNA-V Reverse	5' - ACG UGA CAC GUU CGG AGA ATT - 3'

### Real-Time PCR

Total RNA extraction Kits and PrimerScript® RT reagent Kit with gDNA Eraser (Perfect Real Time) Kits (TaKaRa Biotechnology, Dalian, China) were adapted to reverse-transcript into cDNA from cell and renal tissues samples based on the manufacturer's instructions. Real-time PCR was performed using a TB Green™ Premix Ex Taq™ II Reagent Kit (TaKaRa Biotechnology, Dalian, China). The primer sets used were designed and synthesized by GenePharma (Shanghai, China) and were seen in Table 2. The total reaction volume was 25 μL with 2 μL cDNA in a template. PCR amplification conditions were performed as follows: initial denaturation at 95°C for 3 min followed by 40 cycles of denaturation at 95°C for 12 s; annealing at 62°C for 30 s; and extension at 72°C for 30 s. Fluorescence was detected using the TaKaRa Thermal Cycler Dice® Real Time System. Data analysis based on measurements of the threshold cycle was performed using the 2 ^∧^ (–Δ Δ Ct) method after using the ΔCt method in reference to β-actin (Kauppinen et al., 2013).

**Table 2:** Primer sequences.

**Primers**	**Sequences**	**Length (bp)**	**Tm (°C)**
*Vash1*-F	5' - TGG GAA TTT ACC TCA CCA ACA - 3'	21	57.1
*Vash1*-R	5' - CCA TAG GCC GCC TCA TAG - 3'	18	56.7
*Sirt1*-F	5' - TAC CAG AAC AGT TTC ATA GAG CCA T - 3'	25	59.8
*Sirt1*-R	5' - CAA AAT GTA GAT GAG GCA GAG GTT- 3'	24	59.1
*Hif1a*-F	5' - ACA GGA TTC CAG CAG ACC C - 3'	19	59.0
*Hif1a*-R	5' - GCT GAT GCC TTA GCA GTG GTC - 3'	21	61.3
*Tgfb1*-F	5' - ATG GTG GAC CGC AAC AAC - 3'	18	58.2
*Tgfb1*-R	5' - CCA AGG TAA CGC CAG GAA - 3'	18	56.9
*Smad3*-F	5' - CGA CCA CCA GAT GAA CCA CA - 3'	20	60.0
*Smad3*-R	5' - AGC AGG CCC AGA CAG AAG C - 3'	19	62.3
*Actb*-F	5' - TGA CAG GAT GCA GAA GGA GAT TAC - 3'	24	62.0
*Actb*-R	5' - GAG CCA CCA ATC CAC ACA GA - 3'	20	64.4

### Western Blot

Renal tissues and cells were lysed in RIPA lysis buffer with PMSF protease inhibitors and the supernatant remained after centrifugation. Protein concentrations were analyzed by Pierce™ BCA Protein Assay Kit (Thermo Fisher Scientific, USA) using BSA as a standard control. Samples with 5 × loading buffer added were denatured at 100°C for 10 min. Equivalent samples (40–60 μg/sample) were separated with 10% SDS-PAGE and then transferred to PVDF membranes with Trans-Blot Tank (Bio-rad, USA). Membranes were blocked in TBS-T (Tris buffered saline Tween® 20, pH 7.6; Sigma-Aldrich, Shanghai, China) with 5% BSA 2 h at room temperature, then probed with primary antibodies (Table 3) at 4°C shaking overnight. Then membranes were incubated with a concentration of specific secondary antibody (Table 3) for 2 h after fully washing, and were reacted with Pierce ECL Plus Substrate (Thermo Fisher Scientific, USA). Immunoreactive bands were visualized by DNR Bio-imaging systems MicroChemi 4.2 (Jerusalem, Israel).

**Table 3:** Western Blot Antibody information.

**Usage**	**Antibodies**	**Species**	**Molecular weight (kDa)**	**Catalog**	**Dilutions**	**Manufacture**
Primary antibodies	Anti-rat VASH-1	Goat	44	sc-49777	1:500	Santa Cruz
	Anti-rat VEGF	Rabbit	42/21	sc-152	1:500	Santa Cruz
	Anti-rat CTGF	Rabbit	38	sc-25440	1:500	Santa Cruz
	Anti-rat Fibronectin	Rabbit	220	sc-9068	1:500	Santa Cruz
	Anti-rat Sirt1	Rabbit	120	sc-15404	1:500	Santa Cruz
	Anti-rat HIF1α	Rabbit	132	sc-10790	1:500	Santa Cruz
	Anti-rat pSmad3	Rabbit	52	#9520	1:500	CST
	Anti-rat Smad3	Rabbit	52	#9523	1:500	CST
	Anti-rat TGFβ1	Rabbit	25/12.5	sc-146	1:500	Santa Cruz
	Anti-rat GAPDH	Rabbit	37	sc-25778	1:500	Santa Cruz
Second antibodies	Anti-rabbit IgG-HRP	Goat	–	sc-2004	1:5000	Santa Cruz
	Anti-goat IgG-HRP	Donkey	–	sc-2020	1:5000	Santa Cruz

### Chemiluminescence and ELISA

Samples were measured by water soluble tetrazolium salts-1 (WST-1) visible light and colorimetric method using superoxide dismutases (SOD), glutathione peroxidase (GSH-PX), catalase (CAT), and malondialdehyde (MDA) assay kits (A001-3, A007-1-1, A005 and A003-1, Nanjing Jiancheng Bioengineering Institute, China). Cell supernatant samples were assayed using transforming growth factor-β1 (TGFβ1) and tumor necrosis factor-α (TNFα) (CSB-E04727r and CSB-E11987r, CUSABIO, Wuhan, China) enzyme-linked immunosorbent assay (ELISA) kits. The inter- and intra-assay coefficients of variation were between <8 and 10%, respectively. The absorbance was measured at 450, 405, 412, 450, and 523 nm wavelength, respectively, with a Power Wave XS (Biotek, Winooski, VT, USA).

### Cell Counting Kit-8

After pre-treatment of RMCs cells with trypsin, a cell suspension was prepared using D-MEM medium. A cell counter was used to quantify the cellular concentration. Cells were then inoculated at the ratio of 6 × 10^3^-1 × 10^4^/100 μl/well in a 96-well plate, then cultured at 37°C in 5% CO_2_ for 24 h. According to the manual of cell counting kit-8 (CCK-8, Dojindo, Japan), we added 10 μl/well testing liquid, and continued 2–4 h incubation. Each group was provided with 5 multiple wells, and the absorbance was measured at 450 nm wavelength for subsequent analysis.

### Statistical Analysis

IBM SPSS statistics program (V.17.0, IBM Corp., USA, 2008) was used for data analysis. Data were analyzed in triplicate and expressed as mean ± SD. Differences between the three groups were analyzed by *T*-test and one-way ANOVA, followed by Fisher's Least Significant Difference test for the normally values with equal variances assumed and Tamhane's T2 test values with equal variances not assumed. All *P*-values were marked two-tailed, and *P* < 0.05 was considered of statistical significance.

## Results

### *In vivo* Model Establishment and Low Expression of VASH-1 in Renal Tissues

**Figure 1:**
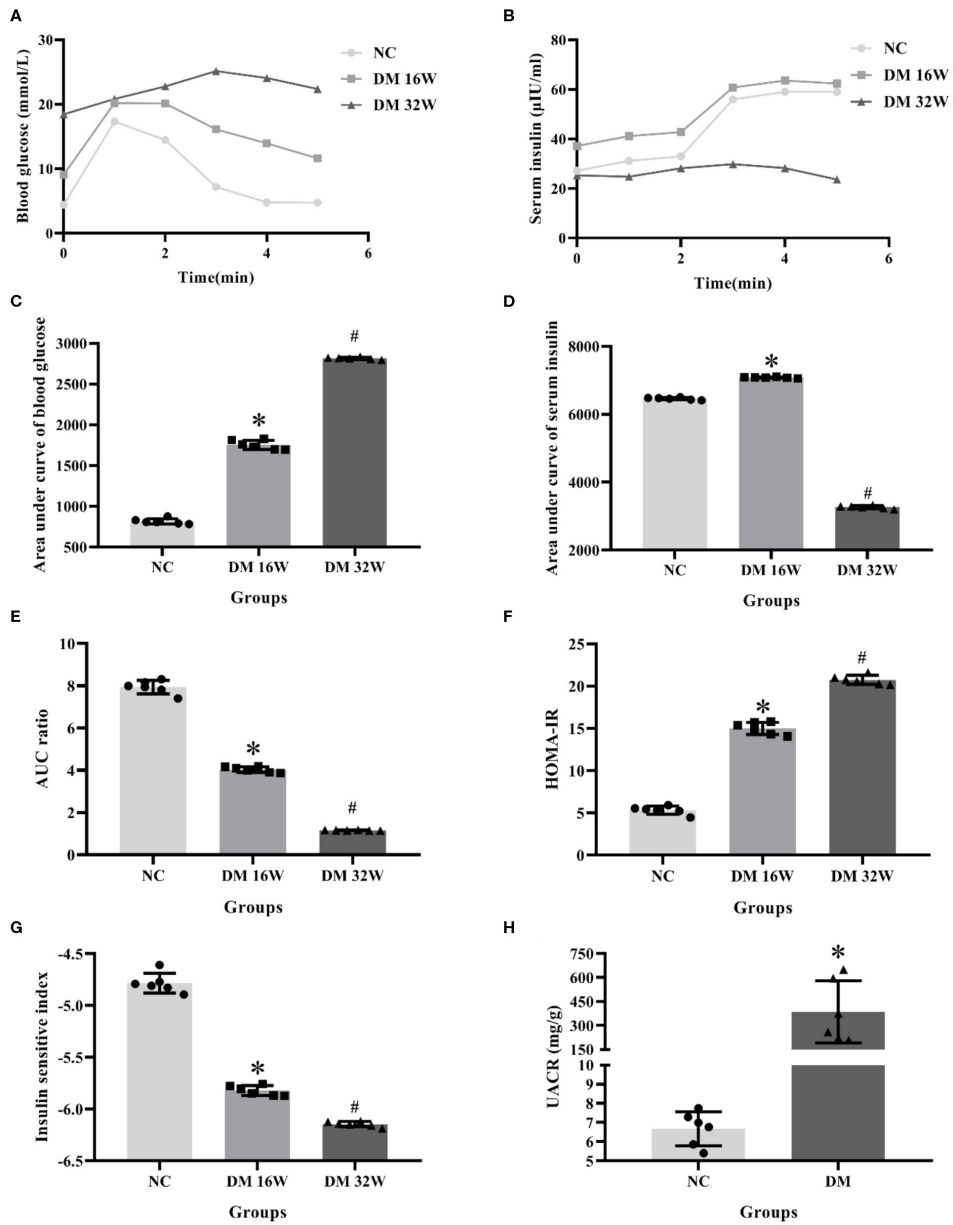
Changes of biological indicators in rat kidney tissues. **(A)** IPGTT test, **(B)** IRT test, **(C)** area under curve of blood glucose (AUC GLU), **(D)** area under curve of serum insulin (AUC INS), **(E)** AUC ratio (AUC INS/AUC GLU), **(F)** HOMA-IR, **(G)** ISI, **(H)** UACR levels. *Compared with the NC group, *P* < 0.05, ^#^Compared with the DM 16W group, *P* < 0.05. Data were expressed as mean ± SD, and experiments were repeated three times.

IPGTT and IRT results showed that compared with the NC group, the blood glucose levels in DM 16 and 32W groups were significantly increased. Serum insulin levels in the DM 16W group were elevated while reduced in DM 32Wgroup. The range of blood glucose was 16.67–25 mmol/L, AUC GLU and HOMA-IR in DM groups were significantly higher, while AUC ratio and ISI lower than that in the NC group, and AUC INS elevated in DM 16W while reduced in DM 32W, consistent with the characteristics of diabetes. Moreover, the UACR in the DM group was significantly elevated compared to the NC group (*P* < 0.05, Figure 1).

**Figure 2:**
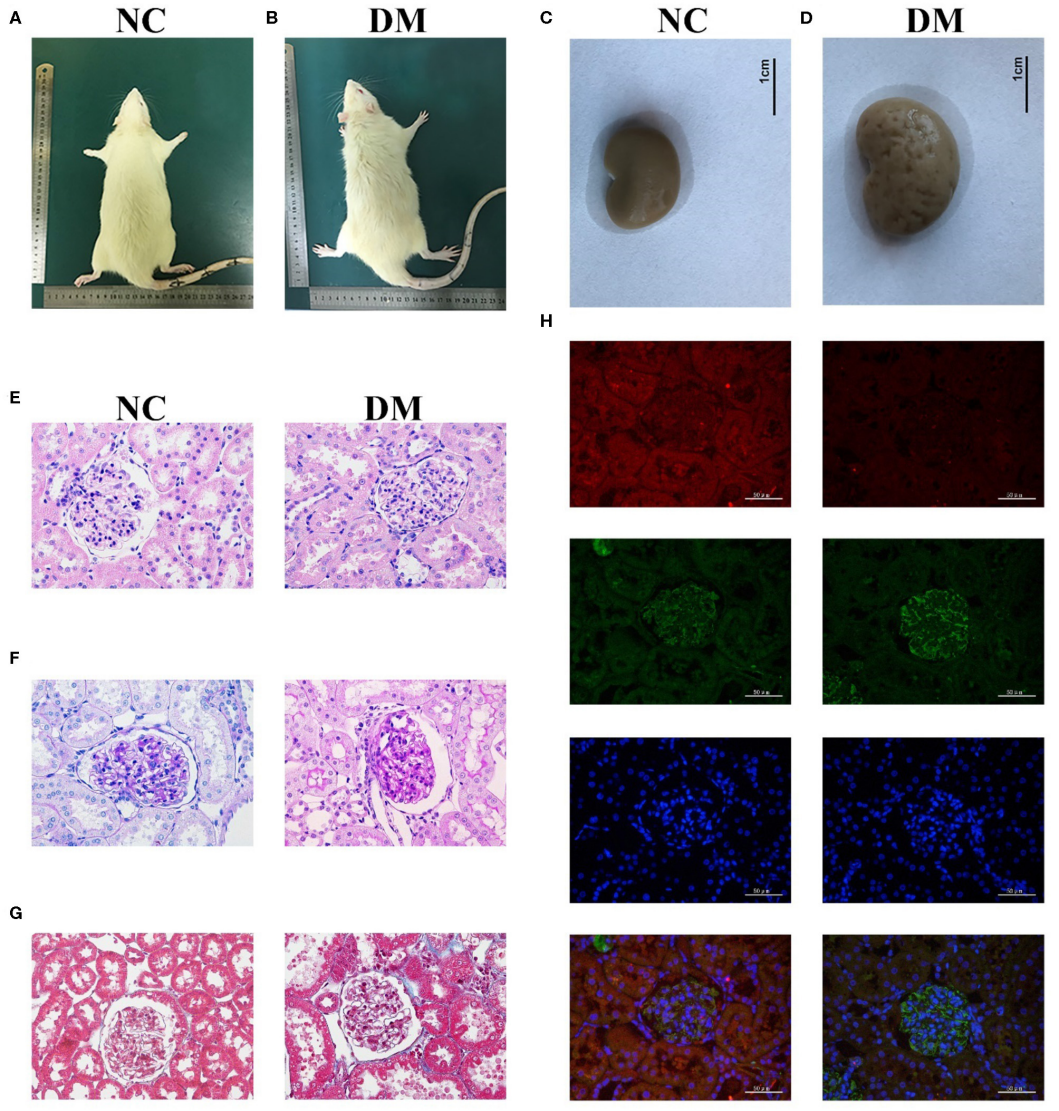
Renal histology in diabetic rats. **(A,B)** photos of rats, **(C,D)** gross specimen, **(E)** HE stain, **(F)** PAS stain, **(G)** Masson stain, **(H)** Immunofluorescence double staining of VASH-1 (red), VEGF (green), DAPI (blue), and merge.

The observation of the rats' condition showed that the rats in NC group had moderate body shape, good mental state, sensitive reaction, and stretched body shape, while rats in DM group had weight loss, listlessness, lethargy, and dorsiflexion (Figures 2A,B). General samples of renal tissues showed that kidneys in NC group were smooth in appearance, moderate in size and ruddy, while ones in the DM group were uneven, enlarged and pale (Figures 2C,D).

Morphological changes to the glomerular and renal tubules in the two groups were detected as well. HE and PAS stains showed that the structure of glomeruli and mesangial cells in renal tissues was confused, and the glycogen concentration was significantly increased in the DM group than that in the NC group (Figures 2E,F). Masson stains showed that the renal tissues structure of the DM group was somewhat blue colored and presented obvious changes than that of the NC group (Figure 2G). Immunofluorescence showed decreased VASH-1 and increased VEGF in renal tissues of the DM group (Figure 2H).

**Figure 3:**
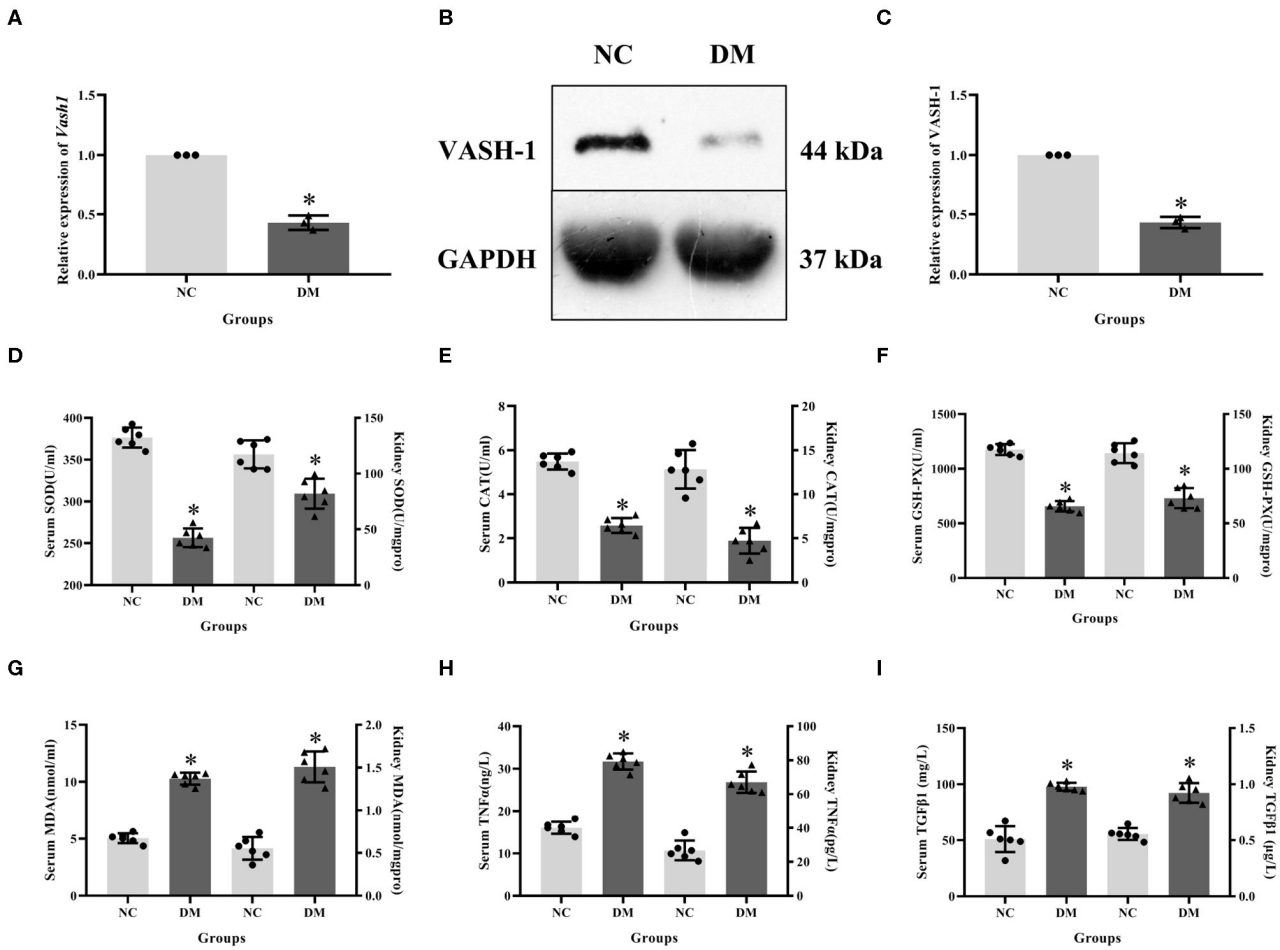
Molecular biology results in diabetic rats. **(A)** relative expression of *Vash1* mRNA, **(B)** western blot protein band, **(C)** relative expression of VASH-1 protein, **(D)** SOD, **(E)** CAT, **(F)** GSH-PX, **(G)** MDA, **(H)** TGFα, **(I)** TGFβ1 in serum and kidney tissues. *Compared with the NC group, *P* < 0.05. Data were expressed as mean ± SD, and experiments were repeated three times.

Expression levels of VASH-1 mRNA and protein in renal tissues in the NC and DM groups were detected. Real-time PCR and western blot results showed that the mRNA and protein levels of VASH-1 in the DM group were significantly reduced than those in the NC group (*P* < 0.05, Figures 3A–C). Compared with NC group, SOD, CAT and GSH-PX in serum and kidney of DM group were significantly decreased, while MDA, TNFα and TGFβ1 increased (*P* < 0.05, Figures 3D–I).

### Effect of High Glucose on VASH-1, Oxidative Stress, and Fibrosis Factors in RMCs

**Figure 4:**
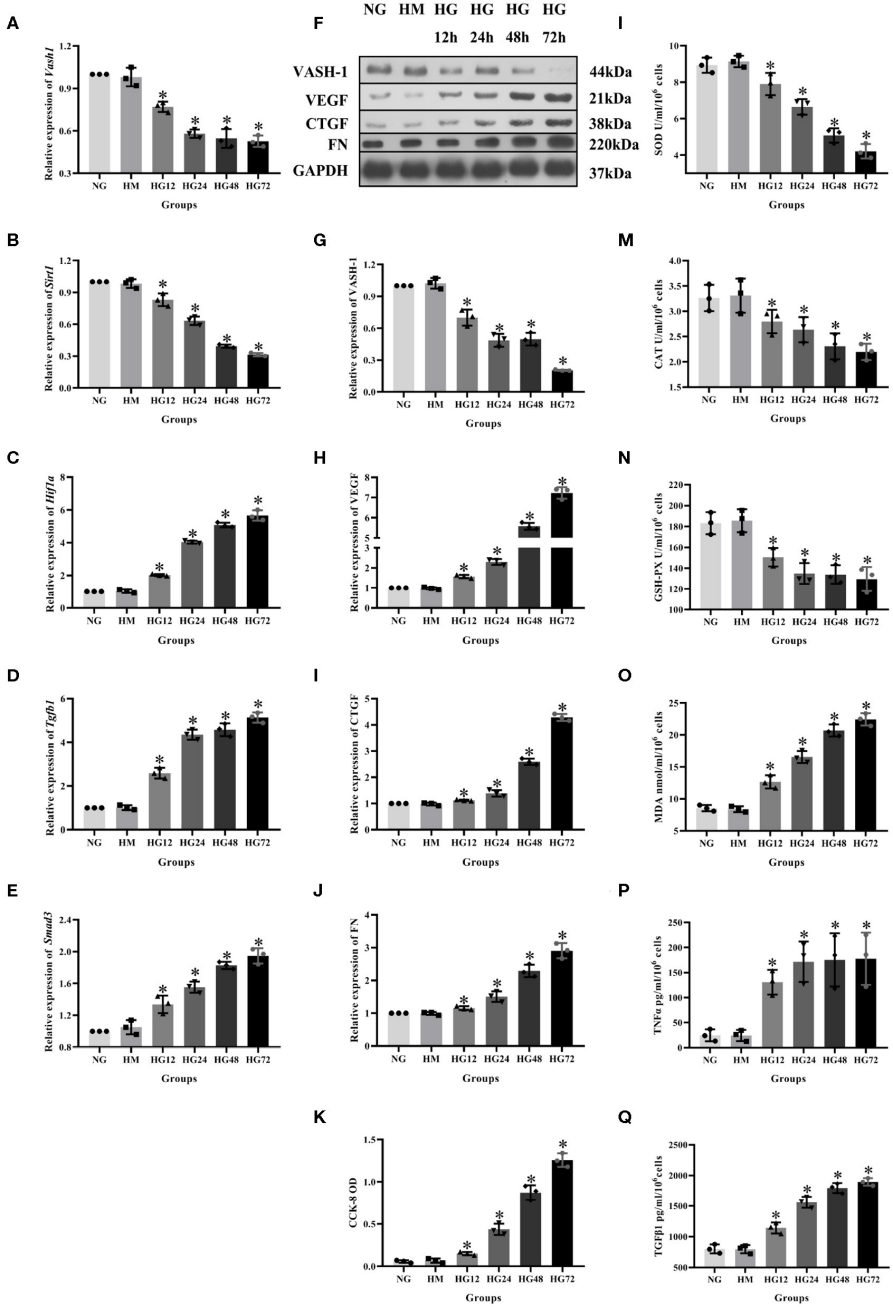
Expression levels of RMCs at different timepoints with high glucose culture. Relative expression of **(A)** *Vash1*, **(B)** *Sirt1*, **(C)** *Hif1a*, **(D)** *Tgfb1*, **(E)** *Smad3* mRNA, **(F)** western blot protein band, relative expression levels of **(G)** VASH-1, **(H)** VEGF, **(I)** CTGF, **(J)** FN protein. **(K)** Detection of RMCs cell proliferation changes in CCK-8. Cell supernatant expression levels of **(L)** SOD, **(M)** CAT, **(N)** GSH-PX, **(O)** MDA, **(P)** TNFα, **(Q)** TGFβ1. *Compared with the NG group, *P* < 0.05. Data were expressed as mean ± SD, and results were repeated three times.

To analyze VASH-1 expression changes in high glucose-cultured RMCs, RMCs were cultured, respectively, in normal glucose, high mannitol control and high glucose (NG, HM and HG) *in vitro*. Meanwhile RMCs with HG was cultured for 12, 24, 48, and 72 h (HG12, HG24, HG48, and HG72). Real-time PCR detection results found that high glucose can inhibit RMCs *Vash1* mRNA expression, and showed time-dependent. *Vash1* expression in high glucose cultured RMCs in 24 h tends to be stable, whose expression levels were approximately equal to 1/2 of that in NG group (*P* < 0.05, Figures 4A–E). Western blot results showed that compared with NG group, VASH-1 protein expression was decreased, while VEGF, CTGF, and FN protein expression increased in HG group, whose change started at 24 h (*P* < 0.05). In addition, there was no statistically significant difference in the expression levels of VASH-1, VEGF, CTGF, and FN between NG and HM groups (*P* > 0.05, Figures 4F–J), thus excluding the influence of osmotic pressure on RMCs *in vitro*. ELISA was used to detect the cells supernatant in each group, the results of which showed that compared with NG group, the expression levels of SOD, CAT, and GSH-PX were decreased, while MDA, TNFα, and TGFβ1 were increased in the HG group (*P* < 0.05), however, there was no significantly difference between NG and HM groups (*P* > 0.05, Figures 4L–Q). CCK-8 was adapted to analyze cell proliferation activity, indicating that compared with NG group, cell proliferation was abnormally increased in HG group (*P* < 0.05), but there was no difference between NG and HM groups (*P* > 0.05, Figure 4K).

### Detection of VASH-1 Transfection Efficiency

**Figure 5:**
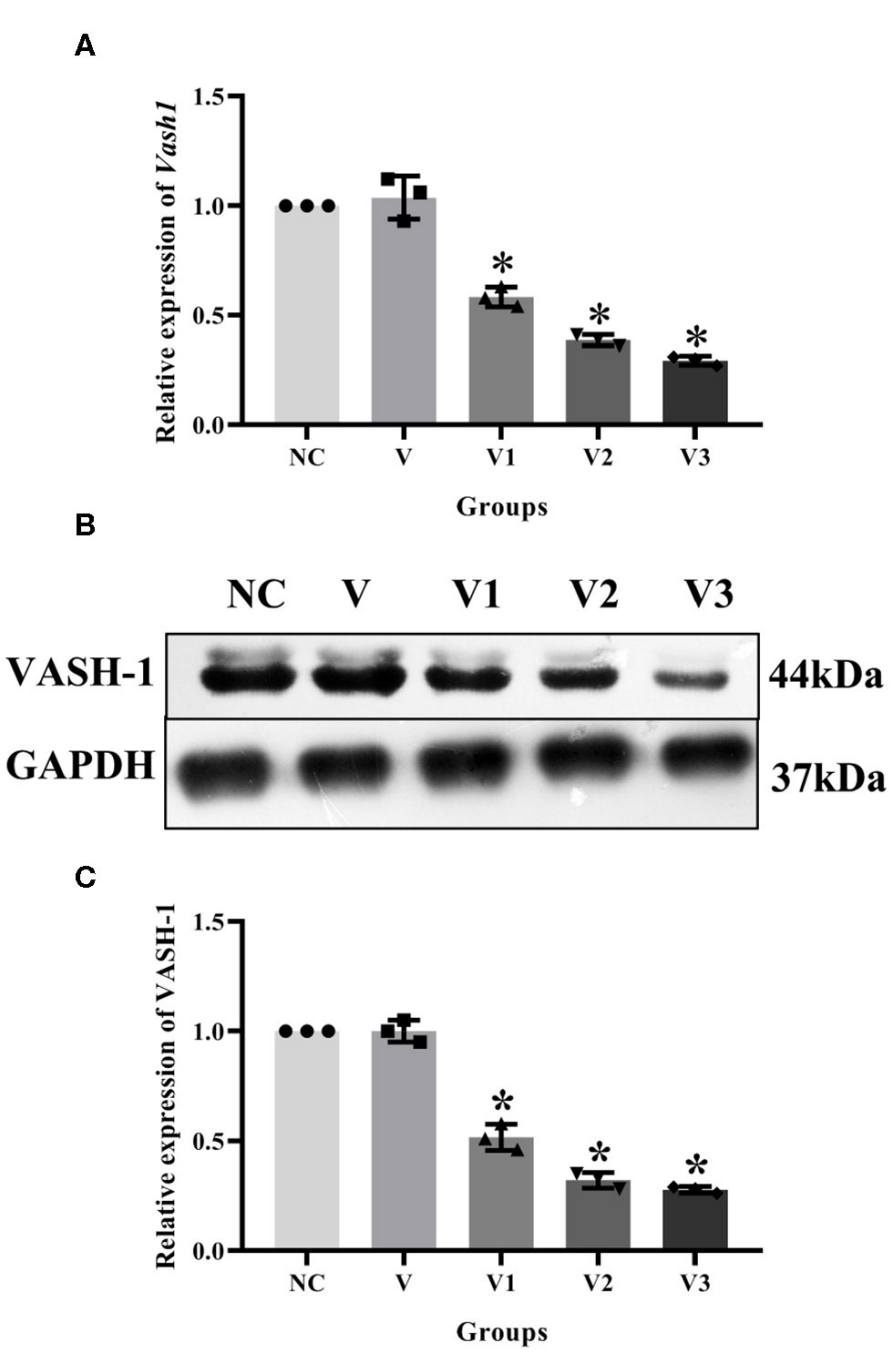
Effects of different *Vash1* siRNA transfection fragments on mRNA and protein expression in RMCs. Relative expression levels of **(A)** *Vash1* mRNA, **(B)** western blot protein bands, and expression levels of **(C)** VASH-1 protein. *Compared with the NC group, *P* < 0.05. Data were expressed as mean ± SD, and results were repeated three times.

In order to detect the transfection efficiency of *Vash1* on high glucose cultured RMCs, gene silencing technology was used to inhibit *Vash1* mRNA. Cells were divided into normal control, small fragment control, small fragment 1, 2, and 3 groups (NC, V, V1, V2, and V3). Real-time PCR and western blot results suggested that the mRNA and protein expression of VASH-1 in the siRNA-V3 transfected group was significantly decreased than others, the inhibition rate of *Vash1* mRNA was 29.3%, and protein was 29.7% (*P* < 0.05, Figures 5A–C). Additionally, the results showed no statistically significant difference in the concentration of *Vash1* mRNA and protein between the NC and V groups (*P* > 0.05), excluding the effect of transfection on RMCs. Therefore, based on the detection results of the transfection efficiency of *Vash1* siRNA, we selected the siRNA-V3 transfection fragment with the highest transfection efficiency as the follow-up study of VASH-1 in RMCs.

### High Glucose and Vash1 siRNA Up-Regulated the Oxidative Stress and Fibrosis Factors

**Figure 6:**
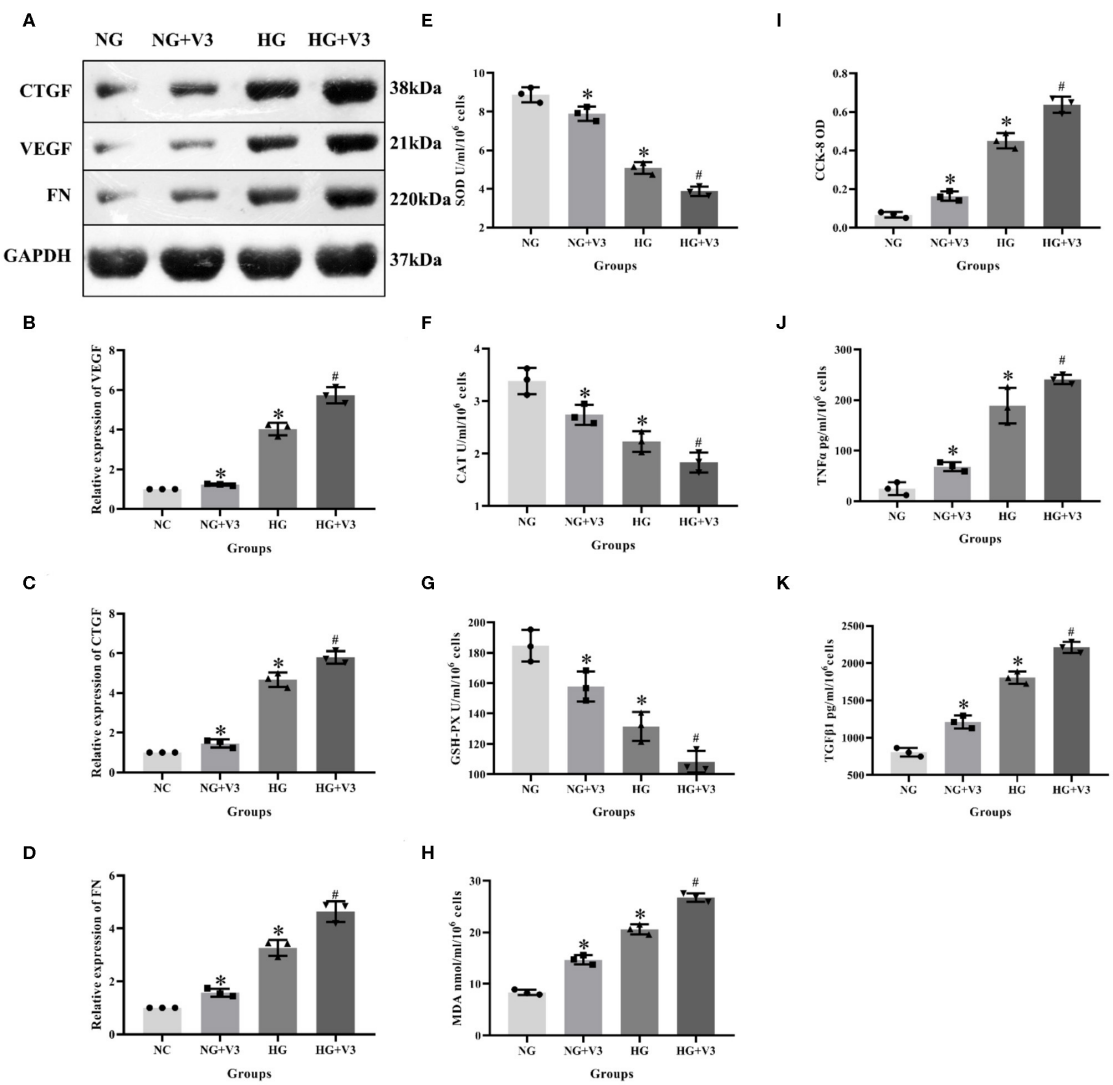
Effects of *Vash1* siRNA transfection in high glucose cultured RMCs. **(A)** Western blot protein bands, relative expression levels of **(B)** VEGF, **(C)** CTGF and **(D)** FN protein, cell supernatant expression levels of **(E)** SOD, **(F)** CAT, **(G)** GSH-PX and **(H)** MDA, **(I)** detection of RMCs cell proliferation changes in CCK-8, cell supernatant expression levels of **(J)** TNFα and **(K)** TGFβ1. *Compared with the NG group, *P* < 0.05; ^#^Compared with the HG group, *P* < 0.05. Data were expressed as mean ± SD, and results were repeated three times.

According to the above transfection efficiency measurement experiment, siRNA-V3 fragments and 24 h incubation time were selected and RMCs were then divided into normal glucose, normal glucose transfection, high glucose and high glucose transfection groups (NG, NG+V3, HG, and HG+V3). Western blot results showed that the expression of fibrosis factors VEGF, CTGF, and FN in the HG group was significantly elevated than that in the NG group, levels of the HG+V3 group was higher than the HG group, and the concentration in the NG+V3 group was increased than that in the NG group (*P* < 0.05, Figures 6A–D). The cell supernatant of each group was detected by ELISA, the results of which indicated that the concentration of MDA, TNFα, and TGFβ1 in the HG group was significantly increased, and SOD, CAT, and GSH-PX reduced than that of the NG group (*P* < 0.05). The same change was observed between HG+V3 and HG, NG+V3 and NG groups (*P* < 0.05, Figures 6E–H,J,K). CCK-8 showed that cell proliferation in the HG group was significantly up-regulated than that in the NG group, and so did HG+V3 and HG, NG+V3 and NG groups (*P* < 0.05, Figure 6I).

### High Glucose and Vash1 Gene Silencing Down-Regulated SIRT1, Up-Regulated HIF1α, TGFβ1, and Smad3

**Figure 7:**
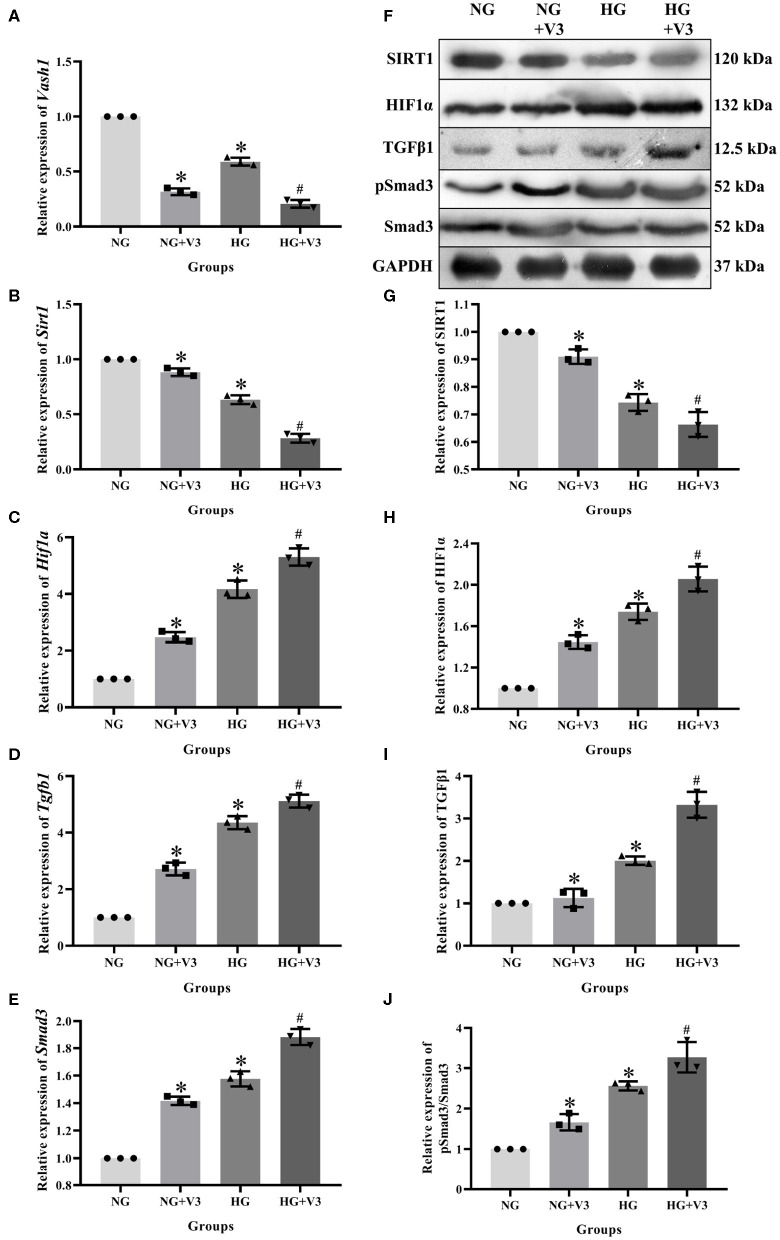
Effects on pathway of *Vash1* siRNA transfection in high glucose cultured RMCs. Relative expression levels of **(A)** *Vash1*, **(B)** *Sirt1*, **(C)** *Hif1a*, **(D)** *Tgfb1*, and **(E)** *Smad3* mRNA, **(F)** Western blot protein bands, relative expression levels of **(G)** SIRT1, **(H)** HIF1α, **(I)** TGFβ1, **(J)** pSmad3/Smad3. *Compared with the NG group, *P* < 0.05; ^#^Compared with the HG group, *P* < 0.05. Data were expressed as mean ± SD, and results were repeated three times.

In order to clarify the effect of pathway in high glucose and *Vash1* gene silencing cultured RMCs *in vitro*, cells were divided into normal glucose, normal glucose transfection, high glucose and high glucose transfection groups (NG, NG+V3, HG, and HG+V3), respectively. Real-time PCR detection found that compared with NG group, high glucose reduced *Sirt1* and increased *Hif1a, Tgfb1*, and *Smad3* mRNA expression. Compared with NG group, *Sirt1* expression was decreased, while *Hif1a, Tgfb1*, and *Smad3* were increased in NG+V3 group. *Sirt1* expression was reduced and *Hif1a, Tgfb1*, and *Smad3* were elevated in the HG+V3 group than that of the HG group. Western blot results consistent with the above results (*P* < 0.05, Figure 7).

### SIRT1 and HIF1α Regulated TGFβ1 and Smad3, Remaining VASH-1 Unchanged

**Figure 8:**
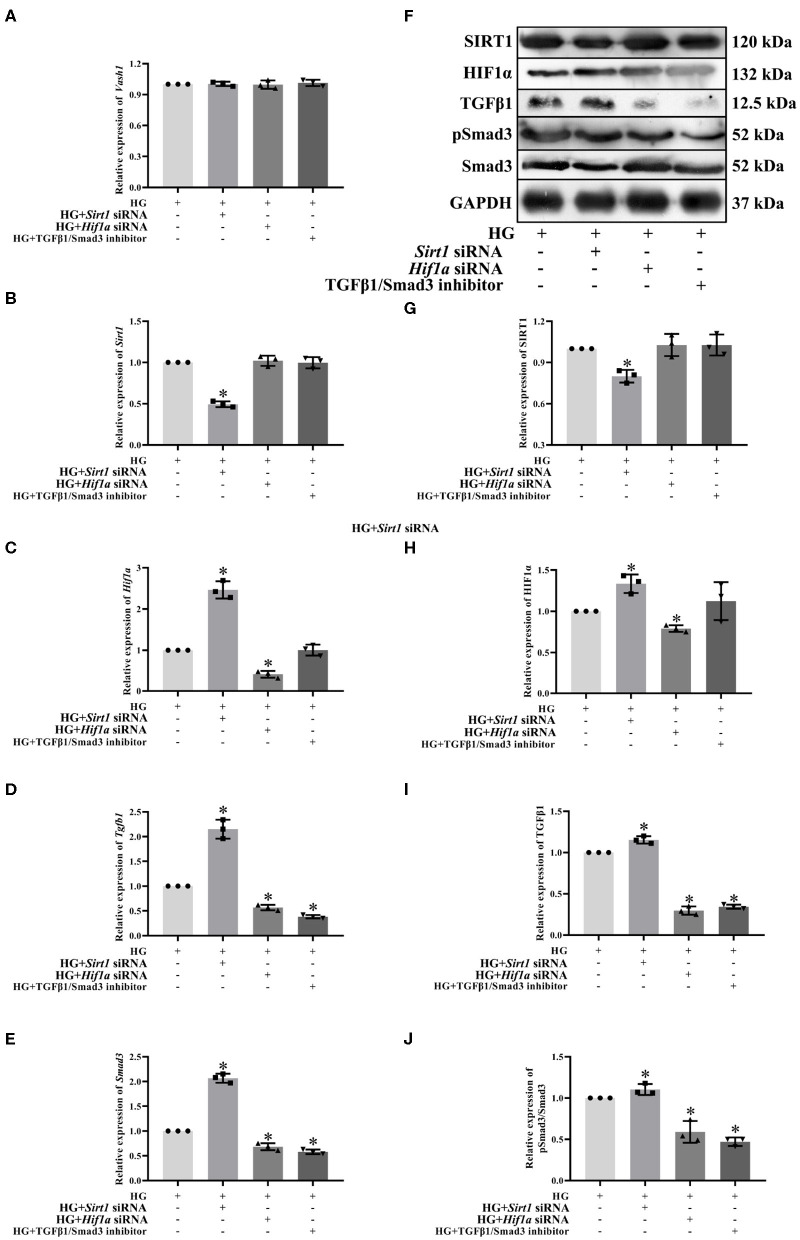
Effect of *Sirt1, Hif1a* siRNA, and the TGFβ1/Smad3 pathway inhibitor on pathway expression in high glucose-cultured RMCs. Relative expression levels of **(A)** *Vash1*, **(B)** *Sirt1*, **(C)** *Hif1a*, **(D)** *Tgfb1*, and **(E)** *Smad3* mRNA, **(F)** Western blot protein bands, relative expression levels of **(G)** SIRT1, **(H)** HIF1α, **(I)** TGFβ1, **(J)** pSmad3/Smad3. *Compared with the HG group, *P* < 0.05. Data were expressed as mean ± SD, and results were repeated three times.

To clarify the regulatory relationship of VASH-1 on SIRT1/HIF1α and TGFβ1/Smad3 signaling pathways in RMCs, cells were divided into high glucose, high glucose *Sirt1* siRNA, high-glucose *Hif1a* siRNA and high-glucose TGFβ1/Smad3 inhibitor groups (HG, HG+ *Sirt1* siRNA, HG+ *Hif1a* siRNA, and HG+ TGFβ1/Smad3 inhibitor). Real-time PCR results showed that there was no significantly difference of *Vash1* among four groups (*P* > 0.05, Figure 8A), indicating that VASH-1 was not affected by SIRT1, HIF1α, or TGFβ1/Smad3 pathway. Compared with the HG group, the relative expression of SIRT1 mRNA and protein was significantly decreased in the HG+ *Sirt1* siRNA group, HIF1α were higher in the HG+ *Sirt1* siRNA group and lower in the HG+ *Hif1a* siRNA group, and TGFβ1 and Smad3 (or pSmad3/Smad3) were elevated in the HG+ *Sirt1* siRNA, and reduced in the HG+ *Hif1a* siRNA and HG+ TGFβ1/Smad3 inhibitor group (*P* < 0.05, Figure 8). However, there was no statistically significant difference in *Sirt1* mRNA and protein among the HG+ *Hif1a* siRNA, HG+ TGFβ1/Smad3 inhibitor, and HG groups, and no difference of HIF1α between HG+ TGFβ1/Smad3 inhibitor and HG groups as well (*P* > 0.05, Figures 8B–J).

## Discussion

Diabetic kidney disease (DKD), a microvascular complication of type 2 diabetes mellitus (T2DM), is also the most common cause of end-stage renal disease (ESRD). Its clinical manifestation is characterized by continuous slow development of proteinuria, eventually leading to kidney failure. Pathological features include glomerular basement membrane thickening, glomerular hypertrophy, abnormal proliferation of mesangial cells, extracellular matrix (ECM) deposition, and in severe cases, glomerular and interstitial fibrosis (Affara et al., 2013; Afkarian et al., 2016). This disease is of great significance for medical research to reduce and delay the occurrence of diabetic kidney damage and explore the possible biological targets.

In order to study the pathological mechanism of DKD, we established a diabetic rat model induced by high-fat diet and low-dose STZ *in vivo*. Results of IPGTT showed that the glucose peak in the DM group was delayed, AUC GLU and UACR were significantly increased compared with the NC group. Histological results showed that the structure of glomeruli and mesangial cells in renal tissues was confused (HE staining), and the glycogen concentration was significantly increased (PAS staining). All of the above changes indicated the successful model establishment of diabetic rats. The levels of VASH-1 were significantly decreased in both molecular biological test and immunofluorescence of renal tissues in diabetic rats, suggesting that VASH-1 may be potentially related to the renal injury mechanism of DKD.

VASH-1 is a novel endothelial angiogenic regulator encoded by highly conserved genes (Kosaka et al., 2013), which are widely present in various kind of tissues and cell lines. The expression levels of brain and placenta were the most abundant, followed by heart and kidney (Kozako et al., 2012). It is produced and secreted in endothelial cells and also expressed in mesangial cells (Bergers and Hanahan, 2008). These proteins exist in a variety of diseases in many models (Lu et al., 2010, 2016; Miyashita et al., 2012; Murakami et al., 2014) where they regulate vascular endothelial growth factors (VEGF) and fibroblast growth factors (FGF) via negative feedback inhibition of endothelial cell migration and proliferation (Akgun and Ertel, 1985; Lu et al., 2010). Experiments have shown that VASH-1 and DKD may have potential biological effects (Lu et al., 2016).

RMCs secret matrix, produce cytokines, devour and clear macromolecular substances and contract similar to smooth muscle cells, whose functions are associated with early DKD filtration functions and consistent to glomerular matrix increase performance in later DKD. RMCs cultured in high glucose are the classical model for DKD researches (Miyashita et al., 2012). In order to further explore the role of VASH-1 in kidney diseases, we detected VASH-1 levels in high glucose cultured RMCs *in vitro* and used mannitol for the osmotic pressure control test. Our results showed that high glucose inhibited VASH-1 mRNA and protein expression with time dependence, supplementing *in vivo* findings. In addition, there was no significant difference in VASH-1 levels between NC and HM group, indicating that the change of RMCs was not induced by osmotic pressure mediated by high glucose, and the influence of osmotic pressure was excluded.

Literature has shown that VASH-1 can regulate VEGF, inhibit pathological angiogenesis of endothelial cells, and regulate the proliferation and migration of endothelial cells (Akgun and Ertel, 1985; Naito et al., 2009; Murakami et al., 2014). VEGF is able to participate in early angiogenesis regulation of DKD, which may be related to various pathological changes such as glomerular hypertrophy, renal interstitial damage, and increased urine protein (Nasu et al., 2009). Studies have shown that the levels of VEGF-A in the renal cortex of diabetic mice and *Vash1* +/– mice has been increased, and *Vash1* gene knockout presented the same phenomenon, leading to the deterioration of DKD (Brownlee et al., 2016). Other studies have also suggested that increased VEGF-A compensated for renal microvascular damage (Onami et al., 2013), indicating that VASH-1 can play a renal protective role by regulating VEGF. Our research found that VEGF was decreased in renal tissue of diabetic rats under immunofluorescence. Compared with NG group, VEGF protein expression and cell proliferation were significantly increased in NG+V3, HG and HG+V3 groups. This indicated that both high glucose and *Vash1* siRNA can affect VEGF expression, and that VASH-1 can regulate the changes of VEGF levels in high glucose cultured RMCs and play a protective role in kidney diseases.

The occurrence and development of DKD are the results of multiple complex factors such as dysregulation of glucolipid metabolism, changes in renal hemodynamics, vasoactive substances, increased angiogenesis, infiltration of inflammatory cells and renal fibrosis (Oza and Kulkarni, 2018; Pellizzon and Ricci, 2018). Oxidative stress and fibrosis are the two main pathological mechanisms of DKD. Enzyme systems (SOD, CAT and GSH-PX) that maintain the metabolic balance of reactive oxygen species (ROS) are important defense mechanisms of the body against oxidative stress. Inadequate ROS scavenging ability of the enzyme system can lead to oxidative injury and MDA production, the final product of lipid peroxidation (Potente et al., 2007). FN is the main component of ECM, CTGF is the downstream effector of fibrosis, while VEGF and TGFβ1 have also been proved as biomarkers of fibrosis (Nasu et al., 2009; Saito et al., 2011). To explore the pathological mechanism of DKD, we performed Masson staining on renal tissues and detected the levels of corresponding factors in RMCs. The results showed that *in vivo* experiments, the renal tissues structure of DM group were somewhat blue colored and presented obvious changes in glomerular and interstitial fibrosis. The expression levels of VEGF, CTGF and FN proteins in high glucose cultured RMCs were increased. Additionally, TGFβ1 levels in the cell supernatant were significantly increased, indicating significant fibrosis changes. In the kidney tissues and serum samples of the DM group as well as the supernatant of RMCs cultured with high glucose, the oxidative stress markers SOD, CAT, and GSH-PX were significantly decreased, while MDA was significantly increased, and CCK-8 showed an abnormal increase in the proliferation of RMCs, indicating the strong oxidative stress reactivity of diabetic kidney tissues and high glucose cultured RMCs. The above experimental results showed obvious oxidative stress and fibrosis changes of renal tissues and RMCs.

Studies on the pathogenesis of VASH-1 in DKD have reported that VASH-1 has a potential therapeutic effect on early DKD fibrosis by inhibiting TGFβ1 levels in renal tissues of type 1 diabetic mice (Sato, 2013). Obese type 2 diabetic mice, after treatment of injecting with coding *Vash1* gene viral vectors, appeared glomerular hypertrophy, glomerular ultrafiltration, proteinuria, and accumulation of mesangial matrix and significantly suppressed collagen type IV. Contrastingly, immunohistochemical displayed TGFβ1 has also been significantly suppressed, proving that VASH-1 has the inhibitory effect of renal interstitial fibrosis in type 2 diabetes (Shao et al., 2016). Further studies have shown that endogenous VASH-1 has potential protective effects on renal interstitial fibrosis by regulating downstream effector molecules, TGFβ1 and the phosphorylation of Smad3 (Shimizu et al., 2005). In *Vash1* deficient mice, signal transduction mediated by TGF-β/Smad was enhanced to regulate renal inflammatory response and fibrosis (Didion and Faraci, 2005; Dei Cas and Gnudi, 2012). We found that compared with the NG group, mRNA and protein expressions of TGFβ1 and Smad3 were increased, while CTGF, FN in RMCs and TNFα, TGFβ1 in cell supernatant were increased in NG+V3 group, and the same phenomenon appeared in HG and HG-+V3 groups, while the concentration of VASH-1 was not affected by TGFβ1/Smad3 inhibitors. Thus, VASH-1 may promote the process of anti-fibrosis and inflammatory response through the TGF-β/Smad signaling pathway.

SIRT1 is a member of the NAD+ terminal dependent deacetylase family, which is widely expressed in mammals. Many reports have shown that SIRT1 is involved in the pathophysiological processes such as antioxidant, anti-aging, anti-inflammatory, anti-fibrosis, autophagy under hypoxia and renal interstitial fibrosis processes. It was shown to play a role in protecting blood vessels in atherosclerosis, diabetic vascular complications and other cardiovascular diseases (Suzuki et al., 1993; Sivaskandarajah et al., 2012; Tanabe et al., 2017). Hypoxia-inducible factor 1α (HIF1α) is a specific combination of erythropoietin genes of low oxygen-sensitive response elements. The HIF1α disorders and overexpression were caused by a lack of oxygen or genetic. It may have a connection with tumor angiogenesis, energy metabolism, cell survival, and invasion in a variety of pathophysiological related processes (Wang et al., 2015). Studies have shown that HIF1α expression was detected in diabetic nephropathy renal tissues (Watatani et al., 2014).

Our previous studies have confirmed that high glucose may regulate the levels of inflammatory responses and fibrosis factors in RMCs through the SIRT1/HIF1α signaling pathway, suggesting that SIRT1/HIF1α pathway can be used as an important pathway for the study of DKD mechanism (Huang et al., 2014). Knockout of *Vash1* in endothelial cells can also reduce the expression of SIRT1, while knockout of *Sirt1* can increase the expression of VASH-1, indicating that VASH-1 is upstream of the axis of VASH-1-SIRT1 (Xu et al., 2012). Endothelial cells contain high expression of SOD2 (Yang et al., 2013), which can protect blood vessels in the physiological process of oxidative stress (Yao et al., 2017). Studies have shown that VASH-1 can improve the tolerance of endothelial cells to pressure by regulating SIRT1 and SOD2, and delay the vascular senescence caused by oxidation (Chen et al., 2015).

In the pathological specimens of renal cell carcinoma, there was a significant negative correlation between VASH-1 and HIF1α (Bergers and Hanahan, 2008). To evaluate human umbilical vein endothelial cells under oxidative stress mediated by hydrogen peroxide, it was found that VASH-1 mediated degradation of HIF1α by proline hydroxylase, indicating that VASH-1 participated in the regulation of oxidative stress mainly induced by HIF1α (Yun-Peng et al., 2013). We found that compared with the NG group, SIRT1, SOD, CAT, and GSH-PX expression were decreased, HIF1α and MDA expression were increased in HG and NG+V3 groups. Compared with the HG group, SIRT1, SOD, CAT, and GSH-PX expression was reduced, while HIF1α and MDA expression were elevated in the HG+V3 group, while the content of VASH-1 was not affected by *Sirt1* and *Hif1a* siRNA. It indicated that VASH-1 may be involved in regulating the oxidative stress in high glucose cultured RMCs through SIRT1/HIF1α signaling pathway.

In addition, SIRT1 can play a protective role in the pathophysiological changes of DKD by regulating the cellular activity under different renal pressures (Zhang et al., 2017), inhibiting the occurrence of oxidative stress in renal tissues (Zhao et al., 2012), and regulating the fibrosis process mediated by the TGF-β/Smad signaling pathway (Ziyadeh, 1993). It was proved that SIRT1 may be an upstream regulator of the TGF-β/Smad signaling pathway. We found that *Sirt1* and *Hif1a* siRNA could significantly affect expression levels of TGFβ1 and Smad3, but TGFβ1/Smad3 inhibitors could not affect the level changes of SIRT1 and HIF1α. This indicates that SIRT1 and HIF1α may exist upstream of the TGFβ1/Smad3 pathway.

**Figure 9:**
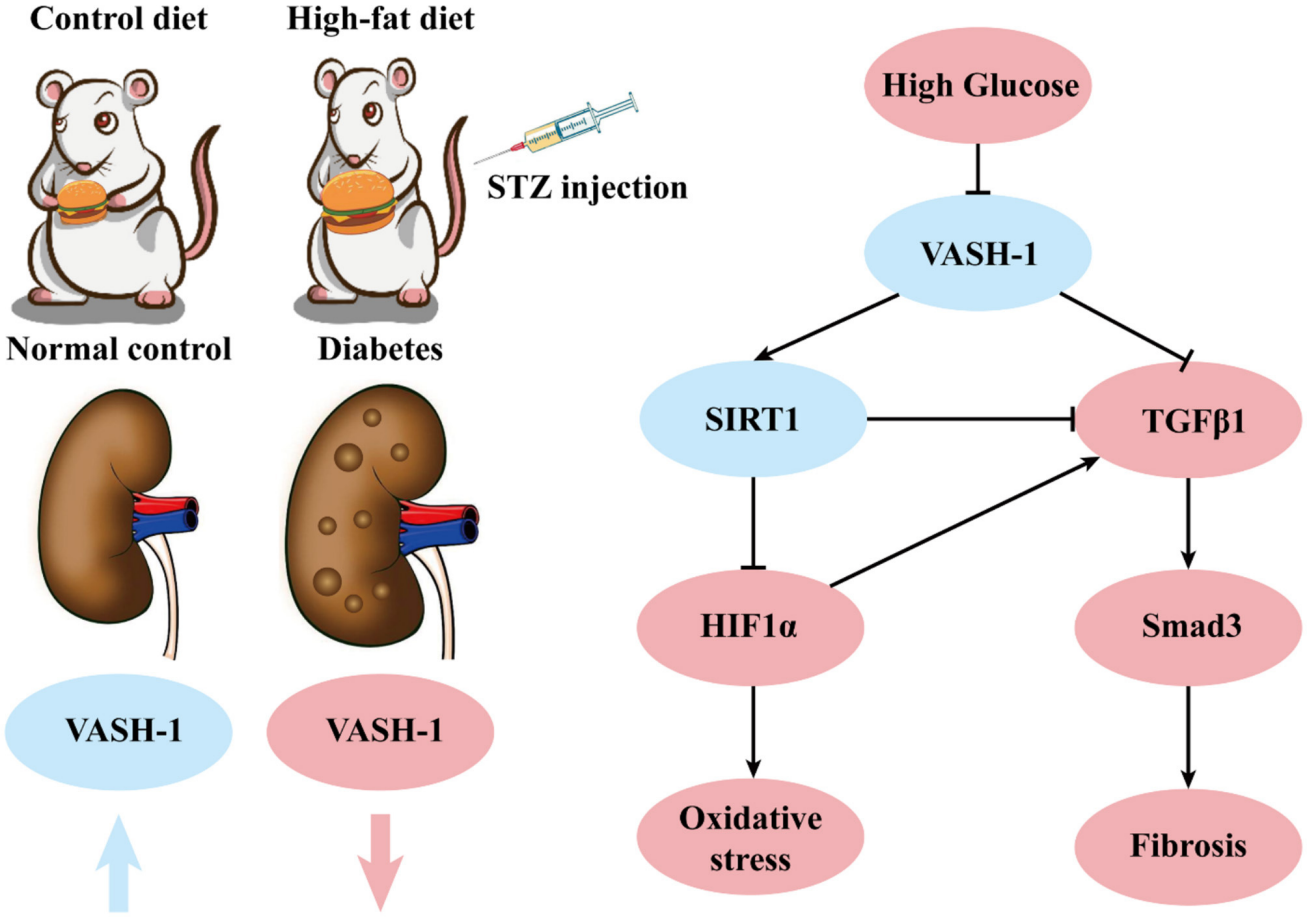
Possible molecular mechanisms.

In summary, VASH-1 expression is suppressed in renal tissues of diabetic rats. As an endothelium-derived angiogenesis inhibitor regulating VEGF, VASH-1 can act on the downstream SIRT1/HIF1α and TGFβ1/Smad3 pathway simultaneously to regulate the pathological process of oxidative stress and fibrosis in DKD (possible molecular mechanism shown in Figure 9).

## Data Availability Statement

All datasets presented in this study are included in the article/supplementary material.

## Ethics Statement

The animal study was reviewed and approved by the Institutional Animal Care and Use Committee (IACUC) of the First Affiliated Hospital of China Medical University (Approval no-2017043).

## Author Contributions

HR: conceptualization, methodology, software, data curation, and writing—original draft preparation. YS, CW, and CL: methodology, data curation, and validation. YZ: software and data curation. QW: conceptualization, reviewing, and editing. All authors contributed to the article and approved the submitted version.

## Conflict of Interest

The authors declare that the research was conducted in the absence of any commercial or financial relationships that could be construed as a potential conflict of interest.
